# Bilateral maxillo-mandibular syngnathia in a newborn

**DOI:** 10.11604/pamj.2014.18.269.5071

**Published:** 2014-08-02

**Authors:** Aziz El Madi, Youssef Bouabdallah

**Affiliations:** 1 Department of Pediatric Surgery, Hassan II University Hospital, Fes, Morocco

**Keywords:** Syngnathia, new-born, maxillo-mandibular, malformation

## Image in medicine

The congenital fusion of the maxilla and mandible is a rare anomaly which is usually diagnosed after birth when it is discovered that the child is unable to open his mouth. Congenital synostosis of the mandible and maxilla is even less common than synechiae, with only 25 cases reported in the literature. We report the case of a five days-old newborn. History of the disease dates back to the birth by finding limiting the opening of the oral cavity with impossibility to breastfeed. The clinical examination of the child on admission to the neonatal department reveal a reactive newborn with birth weight at 2700 g, a good sucking reflex and limited mouth opening (A) with retrognathia (B). No signs of dehydration or malnutrition. Laboratory tests found urea at 1.62 g/l, creatinine at 15 mg/l. renal ultrasound found a right kidney measuring 3.7x 1.6 cm and the left 3.5 x 1.6 cm with well differentiated without dilatation of their pelvis. Maxillary CT with 3D reconstruction revealed hypoplasia of the ascending branches of the mandible; the temporomandibular joints were normal (C,D) with bilateral Maxillomandibular Syngnathia (E,F). A gastric tube was introduced for enteral feeding; the patient gained weight. After general anesthesia; tracheotomy, we proceeded by releasing synechiae between the dental arches and osteotomy of the bone bridge by endobuccal route. A prosthesis was put in place to keep the baby mouth open. The patient was admitted to intensive care where extubation was performed at J2; post-operative follow-up was uneventful.

**Figure 1:**
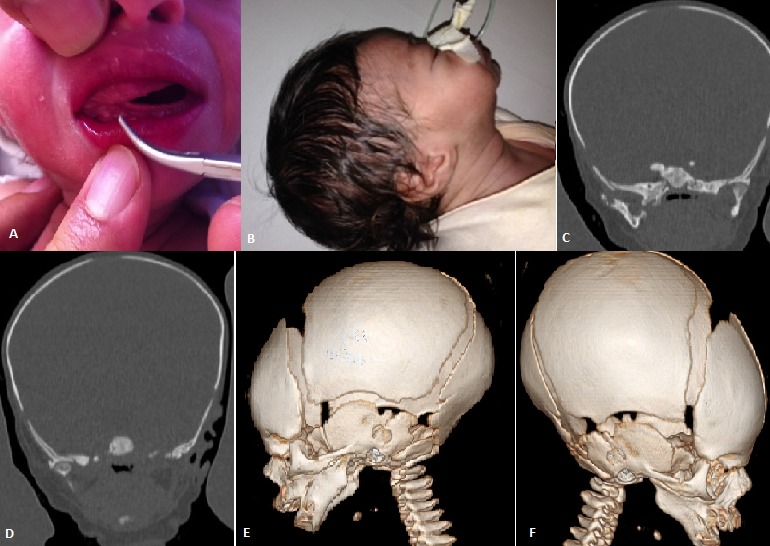
A)opening of the oral cavity limitation; B) retrognathia; C) Coronal CT scan image shows the left temporo-mandibular joint; D) Coronal CT scan image shows the right temporo-mandibular joint; E) Maxillary CT with 3D reconstruction revealed left Maxillomandibular synostosis; F) Maxillary CT with 3D reconstruction revealed right Maxillomandibular synostosis

